# Corn versus Barley in Finishing Diets: Effect on Steer Performance and Feeding Behavior

**DOI:** 10.3390/ani11040935

**Published:** 2021-03-25

**Authors:** Hannah M. DelCurto-Wyffels, Julia M. Dafoe, Cory T. Parsons, Darrin L. Boss, Timothy DelCurto, Samuel A. Wyffels, Megan L. Van Emon, Janice G. P. Bowman

**Affiliations:** 1 Department of Animal and Range Sciences, Montana State University, Bozeman, MT 59717, USA; timothy.delcurto@montana.edu (T.D.); megan.vanemon@montana.edu (M.L.V.E.); jbowman@montana.edu (J.G.P.B.); 2 Northern Agricultural Research Center, Montana State University, Havre, MT 59501, USA; jdafoe@montana.edu (J.M.D.); Cory.Parsons2@chsinc.com (C.T.P.); dboss@montana.edu (D.L.B.); samwyffels@montana.edu (S.A.W.)

**Keywords:** barley, cattle behavior, corn, diet, feedlot performance

## Abstract

**Simple Summary:**

A variety of feedstuffs can be used in beef cattle finishing rations. While corn has traditionally been the most popular feed ingredient in the United States, barley, which is better adapted to the growing and climatic conditions of northern latitudes, can also be utilized. This study evaluated the effects of corn and barley finishing rations on cattle performance and behavior in a feedlot system. Corn-fed steers had greater gains and heavier final live weights than barley-fed steers, however, this tended to be accompanied by greater daily intake. No differences in animal behavior were observed between barley or corn-fed steers. Thus, depending on the difference of costs associated with feeding corn or barley in a given year, barley could be a potential high-quality feed source in beef cattle finishing rations. By having a better understanding of feedlot steer performance and behavior of cattle fed either barley or corn-based diets, producers will be more equipped to make informed nutritional management decisions in feedlot production.

**Abstract:**

This study evaluated the effects of barley and corn finishing rations on feedlot performance and behavior of steers. Feedlot rations in this study were comprised of a main concentrate of either corn or barley. Steers were fed in a GrowSafe system to measure individual animal intake and behavior. Weight gain, average daily gain (ADG), and gain:feed were measured for each steer. Feeding behavior including time spent eating (min/day), visits per day, time per visit (min), eating rate (g/min), intake (kg/day), and intake per visit (g) were measured for each individual. Corn-fed steers had greater ADG (*p* < 0.01) and heavier hot carcass weights (HCW; *p* < 0.01). In addition, corn fed steers had a higher yield grade than barley fed steers (*p* < 0.01). No treatment effects (*p* ≥ 0.11) were observed for time spent eating, visits per day, time per visit, eating rate, intake g/kg body weight, or intake per visit. Although corn-fed steers had a greater ADG and HCW than barley-fed steers, they tended to consume more feed (*p* = 0.06). Depending on the difference of costs associated with feeding corn or barley, barley could be a potential high-quality feed source in beef cattle finishing rations.

## 1. Introduction

Finishing rations for beef cattle often require high amounts of energy to efficiently reach target end points. To accomplish this, a variety of high energy cereal grains may be used in cattle feedlot rations. Corn is the most common feed grain in the United States [1]; however, barley is more widely grown in the Pacific Northwest and Northern Great Plains, as it is more adapted to the growing and/or climatic conditions of the region [2]. This region of the country also accounts for approximately 20% of the U.S. cattle inventory [3]. While barley is already a popular ingredient in Canadian feedlots, the opportunity exists to expand its use in United States beef cattle production systems, particularly in regions where corn production is limited, and barley is widely available.

Previous work has demonstrated that barley and corn have comparative nutritive value [4,5,6]. However, research results regarding animal performance have been inconsistent (average daily gain, ADG; quality grade, QG; and yield grade), where some researchers have found that barley-fed animals had equivalent performance or outperformed corn-fed cattle [7,8,9], while others have found barley-fed steers to have poorer performance than corn-fed animals [10]. Disparities in research results comparing barley and corn-fed cattle in performance trials could partially be attributed to variability in the nutritive value of feed barley [11]. Barley starch is more ruminally digestible than starch from corn [12] with a faster rate of in situ dry matter and starch disappearance [13]. Thus, when barley and corn are similarly processed, the starch and protein from barley is degraded faster and to a greater extent in the rumen than corn [14,15]; which can result in an increased likelihood of digestive disorders such as lactic acidosis and reduced performance [16,17].

Additionally, variation in beef cattle performance may also be contributed to individual feeding behavior [18,19,20]. The digestive differences in barley and corn grains could potentially influence cattle feed behavior and subsequent performance. However, information relating individual feeding behavior of animals on barley or corn-based diets is limited. Understanding the feeding behavior of individual cattle in the feedlot could provide insight into performance differences not always reported in studies based on pen averages.

Although previous research has evaluated the use of corn and barley in finishing beef cattle rations, results of these studies have been inconsistent. Further, there is limited information available relating individual feeding behavior to animal performance with differing basal diets. Therefore, the objective of this study was to evaluate the effects of corn or barley finishing diets on feedlot performance, feed intake behavior and evaluate the relationship of feeding behavior to performance. We hypothesized that barley fed steers would have comparable performance characteristics, but physical and chemical characteristics of grain sources could influence feeding behavior.

## 2. Materials and Methods

Experimental procedures described herein were approved by the Agriculture Animal Care and Use Committee of Montana State University (#2016-AA26). All animals used in this study were provided by the Montana Agricultural Experiment Stations, and the study was conducted at the Northern Agricultural Research Center in Havre, Montana.

For two consecutive years, Angus-based yearling steer calves were fed in a feedlot trial from 27 February 2017 to 12 June 2017 (105 days; 427.3 ± 3.7 kg; n = 48) in year 1, and 26 February 2018 to 11 June 2018 (105 days; 406.8 ± 3.4 kg; n = 47) in year 2. Prior to the study, steers were implanted with a Synovex One Feedlot Implant (Zoetis, Parsippany-Troy Hills, NJ, USA). Upon entry to the feedlot, steers were stratified by body weight and assigned to one of two primary basal grain dietary treatments including: (1) Number 2 feed corn or (2) Hockett barley. Hockett barley is a two-rowed dry-land malting barley often fed to livestock when malting parameters are not met [21]. Both barley and corn were dry-rolled, and diets were formulated based on initial basal grain nutrient analysis and contained 80% grain, 12% barley straw, 3% canola oil, and 5% supplement with the exception of year 2 barley ration that contained 10% supplement due to differences in initial protein content. Supplements consisted of vitamin/mineral packages for feedlot steers and protein sources including wheat middling and canola meal (Table S1). Diets were not balanced to be isocaloric; rations were designed based on percent concentration in an effort to be relevant to a production scenario. Ingredient and nutrient composition of the diets are presented in Table 1. Steers were acclimated to their respective diet for 14-days prior to the start of the data collection period. Steers were fed their respective diets once daily at 0800. Diets were fed to achieve maximum individual intake without excessive feed refusals. When clean bunks were present by mid-day for two consecutive days, rations were increased 0.23 kg per head. Feed refusals were removed weekly. All animals had ad libitum water access for the entirety of the trial.

Steers were fitted with an electronic identification ear tag and were adapted to a GrowSafe system (GrowSafe Systems Ltd., Airdrie, AB, Canada) for 14-days prior to the start of the study. A total of 24 GrowSafe electronic feed bunks (12 per treatment; 1 per 2 steers) were used in this study, each equipped with an antenna to detect animal presence. Load cells measured feed disappearance and neck bars allowed for only one animal to enter the feed bunk at a time. Individual animal intake was continuously recorded via wireless transfer to a data-acquisition computer. The system was monitored daily for unaccounted feed balance. When an unaccounted feed balance or adverse weather event occurred, in which we could not account for 95% of the feed disappearance, the GrowSafe system automatically deemed the 24-h period as failed. Prior validation of GrowSafe has demonstrated that accuracy of dry matter intake was not impacted when up to 30% of the data was missing [22]. In our study, 8.54% of the dry matter intake data failed in year 1 and 10.92% failed in year 2, with an average fail rate of 9.73% across both years of the study.

Initial and final unshrunk weights were obtained on two consecutive days and averaged at the beginning and end of the study period. Additionally, steers were weighed once every 28 days during the study. Performance measurements: ADG and feed efficiency (indexed as the ratio kg weight gain: kg feed intake; G:F) were calculated for each steer (Table S2). In addition, ADG and G:F were adjusted by dressing percentage to calculate carcass adjusted ADG and G:F. Feeding behavior measurements: daily dry matter intake (DMI, kg/day), intake g/kg body weight (BW)/day, visits per day, intake per visit (g), time spent eating (min/day), time per visit (min), and eating rate (g/min) were all calculated from GrowSafe data for each individual steer on a daily basis (Table S3). The length of time between intake readings that constitute a new visit to the feeder was predetermined as 300 s for beef cattle [23,24,25]. Daily intake variation, measured as coefficient of variation (CV, %), was based on daily intake estimates for individual animals. When 70% of all steers were visually estimated to have 1.0 cm of back-fat, steers were transported 13 h (1315 km) and slaughtered after a 12-h rest period at a commercial processing plant. After a 48-h chill at 4 °C carcass measurements (hot carcass weight, HCW; marbling; back-fat; ribeye area, REA; kidney pelvic and heart fat, KPH; and yield grade) were collected by a USDA grader (Table S2).

Carcass and performance measurements were analyzed using generalized linear mixed models including diet as a fixed effect and year as a random intercept. Feeding behavior was also analyzed using generalized linear mixed models including diet as a fixed effect with year and individual steer as random intercepts. Individual steer was used as a random intercept to account for autocorrelation of multiple measurements for each individual. The relationship between individual feeding behavior, performance and carcass characteristics (Table S4) were evaluated using a Pearson product-moment correlation test. Individual steer was considered the experimental unit. An α ≤ 0.05 was considered a significant relationship. A tendency was classified as an α ≤ 0.10. All statistical procedures were conducted in R [26].

## 3. Results

Corn-fed steers had 0.14 kg greater ADG (*p* < 0.01) and heavier final live weight (*p* = 0.02) than barley-fed steers (Table 2). A tendency was observed for corn-fed steers to have greater G:F (*p* = 0.08) than barley-fed steers. Carcass adjusted ADG and G:F were greater for corn-fed steers than barley-fed steers (*p* ≤ 0.02). Hot carcass weights were 16-kg heavier (*p* < 0.01) and tended to have greater back-fat thickness (*p* = 0.06) for steers fed corn compared to steers fed barley. In addition, corn fed steers had a higher yield grade than barley fed steers (*p* < 0.01). However, no differences among treatments were detected for marbling or REA (*p* ≥ 0.19).

No treatment effect was observed for time spent eating, visits per day, time per visit, eating rate, intake g/kg BW, or intake per visit (*p* ≥ 0.11; Table 3). There was a tendency for corn-fed steers to eat more feed per day (*p* = 0.06) than barley-fed steers. Additionally, corn-fed steers had a greater variation in intake (CV, %) compared to barley-fed steers (*p* < 0.01).

When evaluating Pearson’s correlations between feeding behavior and performance variables, eating rate was positively correlated to G:F for both barley and corn-fed steers (*R* = 0.51, *R* = 0.40, respectively; *p* < 0.01; Table 4). Conversely, time spent eating per day was negatively correlated to G:F for steers fed both diets (*R* = −0.38; *p* < 0.01). Additionally, intake per day for barley and corn-fed steers was negatively correlated to G:F (*R* = −0.37, *R* = −0.50; *p* < 0.05) and positively correlated to ADG (*R* = 0.42, *R* = 0.41; *p* < 0.01) and HCW (*R* = 0.49, *R* = 0.63; *p* < 0.01). For both barley and corn-fed steers, intake g/kg BW was negatively correlated to G:F (*R* = −0.48, *R* = −0.65; *p* < 0.01). For barley-fed steers specifically, visits per day was positively correlated to HCW (*R* = 0.30; *p* < 0.05) and yield grade was positively correlated to time per visit (*R* = 0.33; *p* < 0.05) and intake per visit (*R* = 0.34; *p* < 0.05). Additionally, back-fat thickness of barley-fed steers was negatively correlated to intake CV (*R* = −0.43; *p* < 0.01). Corn-fed steers displayed a negative relationship between time per visit and G:F (*R* = −0.28; *p* < 0.05) and a positive relationship between intake per visit and HCW (*R* = 0.44; *p* < 0.01).

## 4. Discussion

Although corn and barley diets were formulated to be isonitrogenous, barley diets had a lower % CP than corn diets, likely due to initial sampling error of the bulk feed delivery. To evaluate if protein differences in our dietary treatments had an impact on energy and protein availability and subsequently daily intakes, we used the Beef Cattle Nutrient Requirements model (Model–BCNRM 2016; [27]) to predict daily intakes based on the nutrient analysis of each diet for each year. For all steers, regardless of treatment, intake was greater than what the BCNRM model predicted (Table 5). The rumen degradable protein balance derived from the BCNRM model suggests that both dietary treatments for both years were in excess of NRC requirements. Additionally, for both years, corn and barley-fed steers had greater average daily intakes than the BCNRM predictions, suggesting the differences in dietary protein levels did not limit daily intake.

As previously noted, research comparing the use of barley and corn in beef cattle diets has yielded inconsistent results. This inconsistency could be due to the biological nature of the grains themselves and the diversity in nutrient value of varying barley varieties [28]. In general, corn is higher in starch compared to barley and is also more likely to escape ruminal digestion and be utilized in the small intestine [12]. Starch digestion in the small intestine is theoretically more energy efficient than ruminal fermentation [29] which might explain why corn-fed steers often have improved performance over barley-fed steers [4]. Still, some barley diets have resulted in performance similar to that of corn-fed cattle [7,8].

Our findings are similar to that of Boss and Bowman [4] as well as Tiffany and Spears [30] where corn-fed cattle had greater ADG and HCW compared to barley-fed cattle. It is commonly accepted that net energy is a better measure of energy availability than total digestible nutrients; however, animal performance from previous work seem to be more related to intake of digestible nutrients than by net energy value, as ADG has been reported to be linearly related to digestible starch intake but not digestible DMI [4]. Presumably, due to corn having higher starch compared to barley, the differences in ADG, HCW and yield grade observed between grain sources in our study may be due to the increased intake of starch by corn-fed cattle. However, it should be noted other studies have reported no differences in carcass characteristics due to grain source [31,32].

Overall, the feeding behavior of corn and barley-fed steers in our study were similar. Other research has reported differences in feeding behavior when steers are fed differing basal diets such as wheat or barley [33]. It has also been reported that when cattle consume increased amounts of barley in the diet, feeding behavior is altered in a manner that reduced risk of acidosis [34]. We did not see these unique differences in feeding behavior between corn and barley-fed steers within our study. When evaluating the relationship of feeding behavior and performance, both barley and corn-fed steers displayed a positive correlation between intake and ADG as well as HCW. However, intake was negatively correlated to G:F, suggesting that steers with greater intakes gain more weight but have lower G:F. Eating rate was positively correlated to G:F for both corn and barley-fed steers which is similar to previous research with barley-fed cattle [19]. Additionally, Schwartzkopf-Genswein and coworkers, [19] reported that steers fed barley-based backgrounding and finishing diets with more variable eating patterns exhibited greater ADG and tended to have greater G:F, however, these relationships were not observed for the barley-fed steers in our study. We did not observe any relationship to frequency of bunk visits to animal growth efficiency, which is similar to Streeter et al. [35] where visits were not reflective of ADG. Additionally, in our study, visits per day was only positively associated with HCW for barley-fed steers. Others have observed positive correlations between average daily bunk attendance and duration with ADG and DMI, which suggests cattle that spend more time at the bunk consume more feed and have higher rates of gain [24,36,37]. However, in our study, time spent at the feeder was only negatively associated with G:F.

## 5. Conclusions

While economic analysis of the use of barley and corn diets for beef cattle is beyond the scope of the current study, it should be noted that feeding behavior was similar across dietary treatments and although differences were observed in performance and carcass characteristics, the differences were relatively small. For example, the difference in fat thickness between treatments was <0.2 cm and differences in yield grade were slight, particularly when you consider that yield grade scores are not rounded up (i.e., a yield grade 3.1 and 3.9 are both considered to be a yield grade 3 [38]). Although corn-fed steers had a greater HCW than barley-fed steers, this tended to be accompanied by greater daily intake. Depending on the difference of costs associated with feeding corn or barley in a given year, barley could be a potential high-quality feed source in beef cattle finishing rations.

## Figures and Tables

**Table 1:** Composition and nutrient content of finishing diets containing corn or Hockett barley as basal grains.

	Year 1		Year 2	
	Barley	Corn	Barley	Corn
Ingredient				
Corn, %	-	80.00	-	75.00
Barley, %	80.00	-	80.00	-
Barley straw, %	12.00	12.00	12.00	12.00
Canola oil, %	3.00	3.00	3.00	3.00
Supplement, % ^1^	5.00	5.00	5.00	10.00
Chemical composition				
Dry matter, %	91.40	91.00	91.40	91.30
Crude protein, %	9.50	11.20	8.80	11.60
Acid detergent fiber, %	18.80	15.40	20.10	11.30
Neutral detergent fiber, %	31.70	26.60	36.60	24.10
Total digestible nutrients, %	69.00	75.00	68.00	76.00
Net energy maintenance, Mcal/kg	0.34	0.39	0.32	0.39
Net energy gain, Mcal/kg	0.22	0.26	0.21	0.26

^1^** The supplement composition was formulated based on initial basal grain nutrient analysis and designed to make diets similar in crude protein, minerals and vitamins (Table S1).

**Table 2:** Performance and carcass characteristics of steers consuming finishing diets containing corn or Hockett barley as basal grains at the Northern Agricultural Research Center.

	Barley	Corn	SEM ^1^	*p*-Value
Performance				
Initial weight, kg	416.36	417.67	10.56	0.79
Final weight, kg	596.45	612.59	15.74	0.02
ADG	1.67	1.81	0.01	<0.01
G:F	0.15	0.16	0.01	0.08
Adjusted ADG ^2^	0.97	1.07	0.06	<0.01
Adjusted G:F ^2^	0.08	0.09	0.01	0.02
Carcass				
Hot carcass weight, kg	347.29	363.19	10.08	<0.01
Marbling ^3^	455.13	478.96	18.60	0.19
12th rib fat, cm	0.94	1.09	0.06	0.06
Ribeye area, cm^2^	87.42	86.17	3.17	0.38
Yield grade ^4^	2.4	2.74	0.11	<0.01

^1^ SEM = standard error of the means. ^2^ Adjusted by dressing percentage. ^3^ Based on USDA quality grade/marbling scores: 200–299 = traces; 300–399 = slight; 400–499 = small; 500–599 = modest; 600–699 = moderate. ^4^ Calculated yield grade = 2.5 + (2.5 × adjusted fat thickness, 12th rib, inches) + (0.0038 × hot carcass weight, pounds) + (0.2 × percentage kidney, pelvic and heart fat) − (0.32 × ribeye area, square inches). ADG, average daily gain; G:F, kg weight gain: kg feed intake.

**Table 3:** Feeding behavior of steers consuming finishing diets containing corn or Hockett barley as basal grains at the Northern Agricultural Research Center.

	Barley	Corn	SEM ^1^	*p*-Value
Time spent eating, min/day	105.99	107.92	2.87	0.58
Visits per day	16.40	15.83	0.54	0.14
Time per visit, min	1.38	1.37	0.08	0.94
Eating rate, g/min	101.10	104.38	11.66	0.45
Intake per day, kg *	11.30	11.72	0.52	0.06
Intake g/kg BW *	21.91	22.46	0.24	0.11
Intake per visit, g *	400.70	412.14	15.18	0.34
Intake CV, % ^2^	20.38	24.20	1.59	<0.01

^1^ SEM = standard error of the means. ^2^ CV, coefficient of variation. BW, body weight * Intake reported on a dry mater (DM) basis.

**Table 4:** Pearson correlation coefficients for pair-wise associations between feeding behavior, performance (gain to feed, G:F; average daily gain, ADG) and carcass characteristics (hot carcass weight, HCW; yield grade, YG) of steers consuming finishing diets containing corn or Hockett barley as basal grains at the Northern Agricultural Research Center.

	Barley					Corn				
	G:F	ADG	HCW	YG	FAT	G:F	ADG	HCW	YG	FAT
Time per day	−0.38 **	−0.04	0.01	0.19	0.17	−0.38 **	<0.01	0.03	0.1	0.19
Visits per day	−0.11	0.05	0.3 *	−0.21	0.01	−0.16	0.03	0.07	0.11	0.1
Time per visit	−0.25	−0.09	−0.22	0.33 *	0.16	−0.28 *	−0.03	−0.02	0.02	0.14
Eating rate	0.51 **	0.14	0.31	−0.28	−0.12	0.40 **	0.2	0.17	−0.02	−0.07
Intake per day	−0.37 *	0.42 **	0.49 **	0.22	0.19	−0.50 **	0.41 **	0.63 **	0.23	0.23
Intake per visit	−0.15	0.23	0.08	0.34 *	0.15	−0.28	0.27	0.44 **	0.09	0.1
Intake g/kg BW ^1^	−0.48 **	0.13	−0.16	0.12	0.11	−0.65 **	0.01	0.02	0.13	0.11
Intake CV ^2^	0.23	−0.15	−0.26	−0.26	−0.43 **	−0.10	−0.15	−0.10	−0.24	−0.11

^1^ BW, Body weight. ^2^ CV, coefficient of variation. * Significant associations *p* ≤ 0.05. ** Significant associations *p* ≤ 0.01.

**Table 5:** Observed average daily intake (ADI) and BCNRM predicted ADI, rumen degradable protein (RDP) requirements, available RDP and RDP balance for steers consuming finishing diets containing corn or Hockett barley as basal grains at the Northern Agricultural Research Center.

	Year 1		Year 2	
	Barley	Corn	Barley	Corn
Observed ADI, kg	11.53	12.24	10.69	10.81
BCNRM ^1^ predicted ADI, kg	9.13	8.40	9.29	8.28
RDP required kg/day	0.80	0.93	0.73	0.83
RDP available kg/day	1.20	1.51	1.03	1.37
RDP balance kg/day	0.40	0.58	0.30	0.54

^1^ Model–BCNRM, Beef Cattle Nutrient Requirements Model [27].

## Data Availability

The data presented in this study are available in Supplementary Material Tables S2–S4.

## Supplementary Materials

The following are available online at https://www.mdpi.com/2076-2615/11/4/935/s1, Table S1: Dietary Supplement Composition, Table S2: Animal Performance and Carcass Data, Table S3: Animal Feeding Behavior Data, Table S4: Animal Performance and Feeding Behavior Data for Correlations.
