# Supplementary material for: Corn versus Barley in Finishing Diets: Effect on Steer Performance and Feeding Behavior
Source: Animals (Basel). 2021 Mar 25;11(4):935. doi: 10.3390/ani11040935 (PMC8064474; doi:10.3390/ani11040935)
Supplement: Supplementary file 1 [file animals-11-00935-s001.zip › Appendix S1.pdf]

Appendix S1. Composition and nutrient content of supplement for corn and barley diets

	Year 1		Year 2	
	**Barley**	**Corn**	**Barley**	**Corn**
Ingredient, %				
Wheat Midds	41.86	10.00	42.29	41.40
Canola Meal 34%	-	24.32	-	12.25
Soybean Hulls	-	-	25.00	-
Calcium Carbonte	17.95	15.35	20.00	5.40
Malt Sprouts	15.00	15.00	-	15.00
Meat & Bone Meal – Pork	6.85	15.00	-	15.00
Urea	5.15	8.65	-	6.85
Molasses, Cane	5.00	-	5.00	-
Salt, Bulk	3.52	3.59	4.00	2.02
Feather Meal	-	2.50	-	-
Potassium Chloride	2.17	2.23	2.08	-
Calcium Sulfate	0.87	1.73	-	1.53
Monensin 90 g/kg	0.73	0.73	0.73	0.37
Tylan 40 g/kg	0.51	0.51	0.51	0.24
Chemical composition				
Dry Matter, %	92.65	93.96	90.66	92.48
TDN Rumen, %	46.98	45.39	48.51	57.47
Crude protein, %	30.13	50.25	10.22	43.27
NPN, %	14.83	24.91	-	19.97
Crude Fat, %	2.88	3.40	1.91	3.87
CR Fiber, %	5.81	5.99	12.99	7.29
Acid detergent fiber, %	7.25	8.82	14.77	9.82
Neutral detergent fiber, %	23.47	20.86	29.46	28.93
Calcium, %	8.25	8.24	8.31	4.10
P-Total, %	0.73	1.10	0.39	1.20
Totl Salt, %	3.75	3.75	4.19	2.10
Sodium, %	1.68	1.71	1.66	1.06
Chloride, %	3.36	3.37	3.55	1.37
Potassium, %	2.00	2.00	2.00	1.01
Magnesium, %	0.22	0.27	0.22	0.28
